# Systemic air embolism causing acute stroke and myocardial infarction after percutaneous transthoracic lung biopsy – a case report

**DOI:** 10.1186/s13019-016-0478-z

**Published:** 2016-05-06

**Authors:** Rafael Rehwald, Alexander Loizides, Franz J. Wiedermann, Astrid E. Grams, Tanja Djurdjevic, Bernhard Glodny

**Affiliations:** Department of Radiology, University Hospital for Radiology, Medical University of Innsbruck, Anichstraße 35, A-6020 Innsbruck, Austria; Department of Surgery, University Hospital for Anesthesia and Intensive Care, Medical University of Innsbruck, Anichstraße 35, A-6020 Innsbruck, Austria; Department of Radiology, University Hospital for Neuroradiology, Medical University of Innsbruck, Anichstraße 35, A-6020 Innsbruck, Austria

**Keywords:** Air embolism, Transthoracic biopsy, Stroke, Myocardial infarction, Intensive care

## Abstract

The air embolism in this case was likely to have been caused by positioning the patient in a prone position, which was associated with the lesion to be biopsied being at a maximum height over the left atrium. Due to the resulting negative pressure, air entered through a fistula that formed between the airspace and the pulmonary vein. The air could have been trapped in the left atrium by positioning the patient in left lateral position. The event itself could have been prevented by positioning the patient in an ipsilateral dependent position during the biopsy. In addition to hyperbaric oxygen therapy, the preferred treatment options are positioning maneuvers, administration of pure oxygen, and heparinization.

## Correspondence

### Dear Sir,

**Fig. 1:**
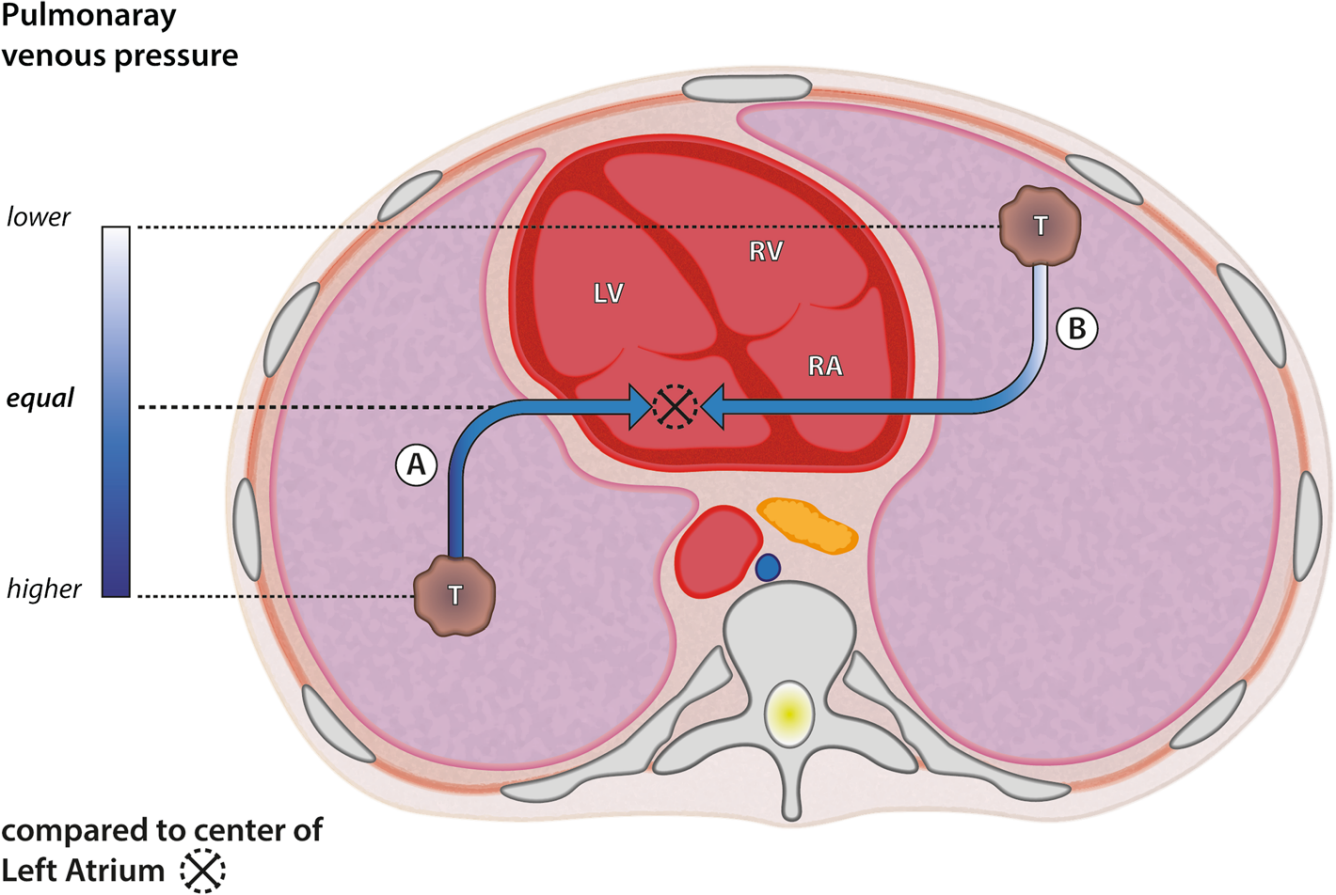
Pulmonary venous pressures depending on the position of the lesion in relation to the left atrium. If the lesion is located below the level of the atrium, the pulmonary venous pressure at this location is the existing pressure in the left ventricle plus the hydrostatic pressure over the lesion to be biopsied (“T”), defined as the distance from the center of the left atrium to the lesion, marked “A” in the figure. If a pulmonary vein is injured, bleeding occurs here. If the lesion is located above the level of the atrium, the pulmonary venous pressure at this location is the existing pressure in the left atrium minus the hydrostatic pressure below the lesion to be biopsied, defined as the distance from the center of the left atrium to the lesion, marked “B” in the figure. If a pulmonary vein is injured, air enters the pulmonary vein due to the lower viscosity of air compared with blood. LV: Left ventricle; RV: right ventricle; RA: right atrium

We read with interest the report by Hung et al. [1] describing the case of a 63-year-old patient who suffered an acute stroke of the left middle cerebral artery and a non-ST elevation myocardial infarction due to an air embolism after a biopsy of a tumor in the lower lobe of the left lung. Although a very large amount of air had entered, filling the aorta to almost 40 % (Fig. 1b [1]), the patient fortunately survived the event and according to the report, was discharged to care at home 7 days later with residual hemiplegia on the right side and presumably persisting global aphasia.

A few remarks are necessary, both regarding the risk of this event and its treatment.

The biopsy was performed in prone position [1], one of the main risk factors for an air embolism [2]. As Fig. 1a shows, the lesion was in the posterior basal segment of the left lower lobe, at the time of the biopsy far above the level of the left atrium, not “under the level of the left atrium“, as the authors describe [1]. In this case, the prone position of the patient placed the lesion as far as possible above the left atrium. This is associated with negative intrapulmonary venous pressure that allows air to enter if an alveolar to pulmonary venous fistula, a bronchial to pulmonary venous fistula, or a direct connection between the tip of the needle and the pulmonary vein occurs. Figure 1 illustrates how the “position of the lesion above or below the level of the left atrium” should be understood. If the patient had been positioned in “ipsilateral dependent position” [3] i.e. in a supine position with the right side elevated somewhat, this complication may not have occurred.

From Fig. 1c [1], which like 1a and 1b is shown laterally reversed, but correctly labeled, it can be seen that a considerable amount of air was in the left atrium of the heart. This air could have been trapped in the left atrium by positioning the patient on the left side [4]. As can be seen in Fig. 1d [1], the patient was presumably turned over his right side onto his back after the biopsy, so the stroke occurred in the left medial cerebral artery. As both the desired and initially existing Trendelenburg positioning of the patient and the left lateral position that would have been protective in this case were abandoned, immediate transport to a pressure chamber should have been considered.

We agree in principle with the risk factors described [1, 2], but would like to note that Hiraki et al. [5] described no increased risk associated with the biopsy of a rather “centrally located lesion” [1] and that in 1990, CT fluoroscopy was not available to Worth et al. [6]. Of course the needle should not be placed directly in a central pulmonary vein and of course it is extremely important to monitor the needle position in real time if possible. However, in order to avoid mid-sized or small pulmonary veins, we recommend setting a thinner collimation than the 5 mm presented in this case [1].

Conservative treatment with aspirin is understandable considering its indication to prevent the development of microthrombi via the tiny arterial air emboli. In this case, however, considering the risk of hemorrhagic transformation of the middle cerebral artery stroke, it would have been preferable to administer heparin because, unlike aspirin, it can be antagonized. In general, administration of 100 % oxygen, not 50 %, is recommended, on the one hand to minimize the size of the gas bubbles by eliminating nitrogen from them and on the other hand to ensure the greatest possible oxygenation of the tissue [7]. Under this treatment, it would have been possible to await the physical resorption of the air in the Trendelenburg position or in left lateral position.

## References

[1] Hung WH, Chang CC, Ho SY, Liao CY, Wang BY (2015). Systemic air embolism causing acute stroke and myocardial infarction after percutaneous transthoracic lung biopsy-a case report. J Cardiothorac Surg..

[2] Freund MC, Petersen J, Goder KC, Bunse T, Wiedermann F, Glodny B (2012). Systemic air embolism during percutaneous core needle biopsy of the lung: frequency and risk factors. BMC Pulm Med..

[3] Rott G, Boecker F (2014). Influenceable and Avoidable Risk Factors for Systemic Air Embolism due to Percutaneous CT-Guided Lung Biopsy: Patient Positioning and Coaxial Biopsy Technique-Case Report, Systematic Literature Review, and a Technical Note. Radiol Res Pract..

[4] Kok HK, Leong S, Salati U, Torreggiani WC, Govender P (2013). Left atrial and systemic air embolism after lung biopsy: importance of treatment positioning. J Vasc Interv Radiol.

[5] Hiraki T, Fujiwara H, Sakurai J, Iguchi T, Gobara H, Tajiri N (2007). Nonfatal systemic air embolism complicating percutaneous CT-guided transthoracic needle biopsy: four cases from a single institution. Chest.

[6] Worth ER, Burton RJ, Landreneau RJ, Eggers GW, Curtis JJ (1990). Left atrial air embolism during intraoperative needle biopsy of a deep pulmonary lesion. Anesthesiology.

[7] Lederer W, Schlimp CJ, Glodny B, Wiedermann FJ. Air embolism during CT-guided transthoracic needle biopsy. BMJ Case Rep. 2011;2011. doi:10.1136/bcr.04.2011.4113 10.1136/bcr.04.2011.4113 PMC3132630 22693299

